# Supplementary figures and images for: RNAi-Mediated Manipulation of Cuticle Coloration Genes in Lygus hesperus Knight (Hemiptera: Miridae)
Source: Insects. 2022 Oct 27;13(11):986. doi: 10.3390/insects13110986 (PMC9698757; doi:10.3390/insects13110986)

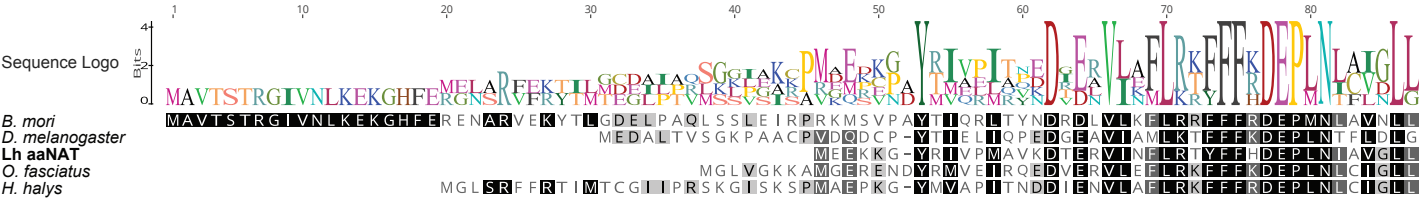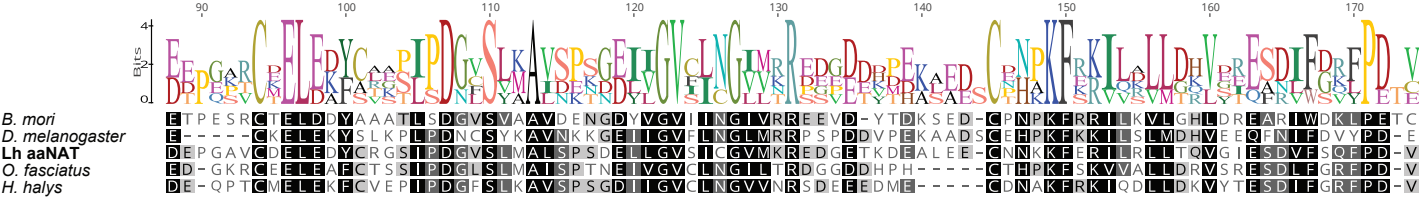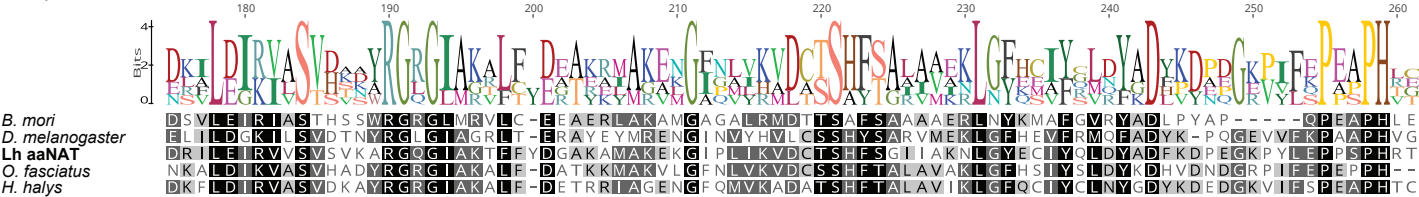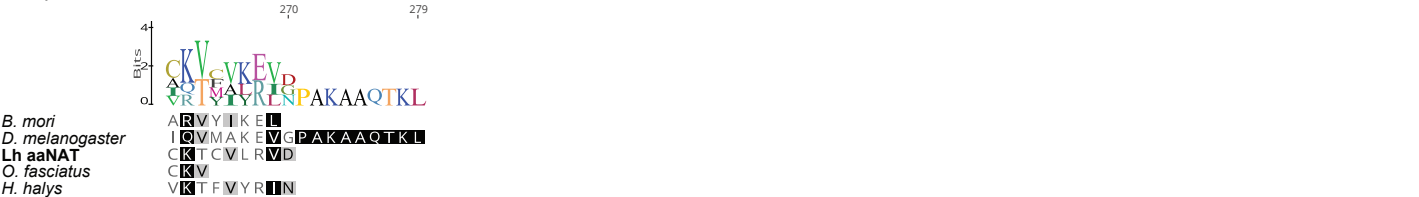

Supplement: Supplementary file 1 [file insects-13-00986-s001.zip › Figure S1 aaNAT.pdf]

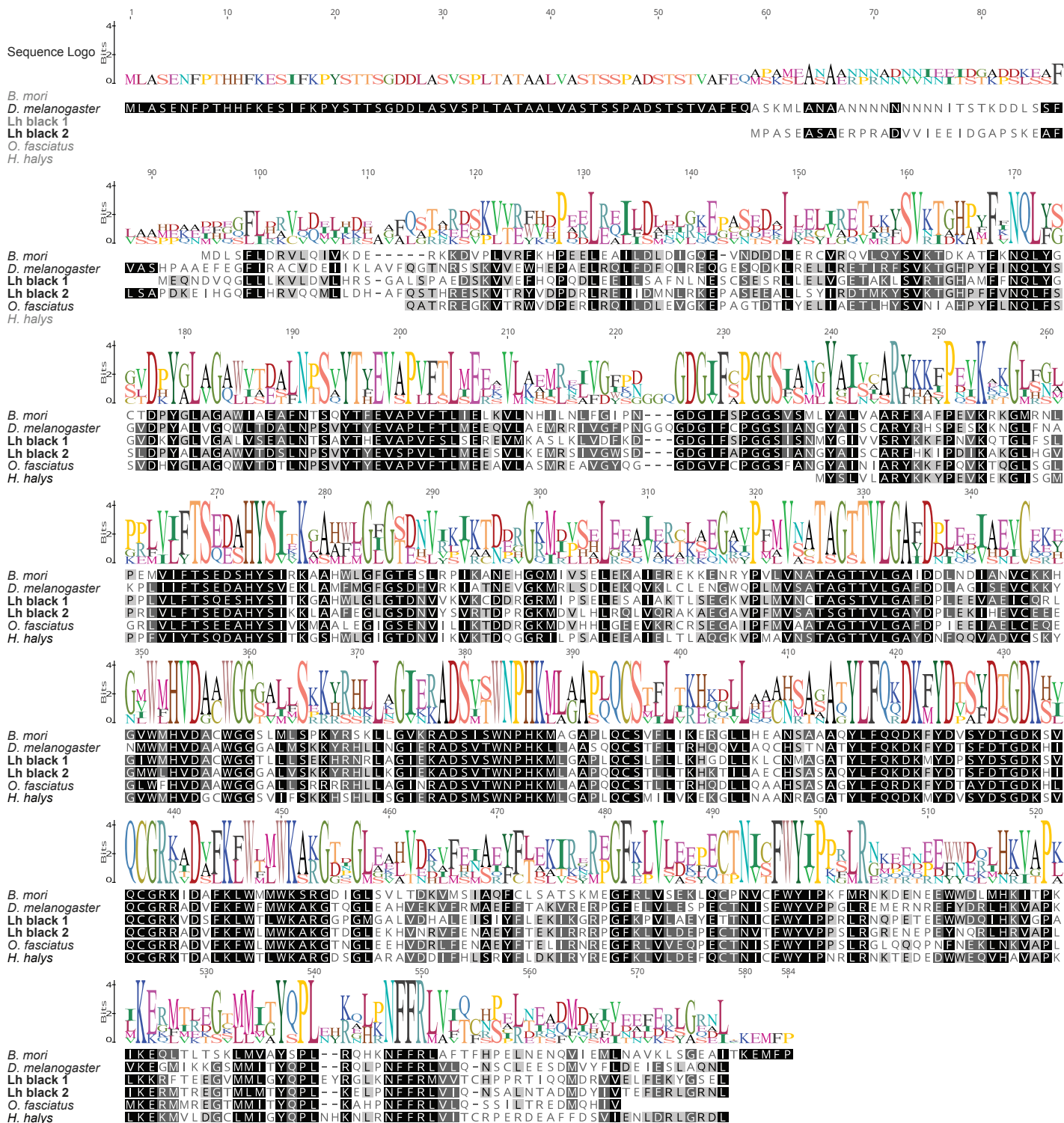

Supplement: Supplementary file 1 [file insects-13-00986-s001.zip › Figure S2 black.pdf]

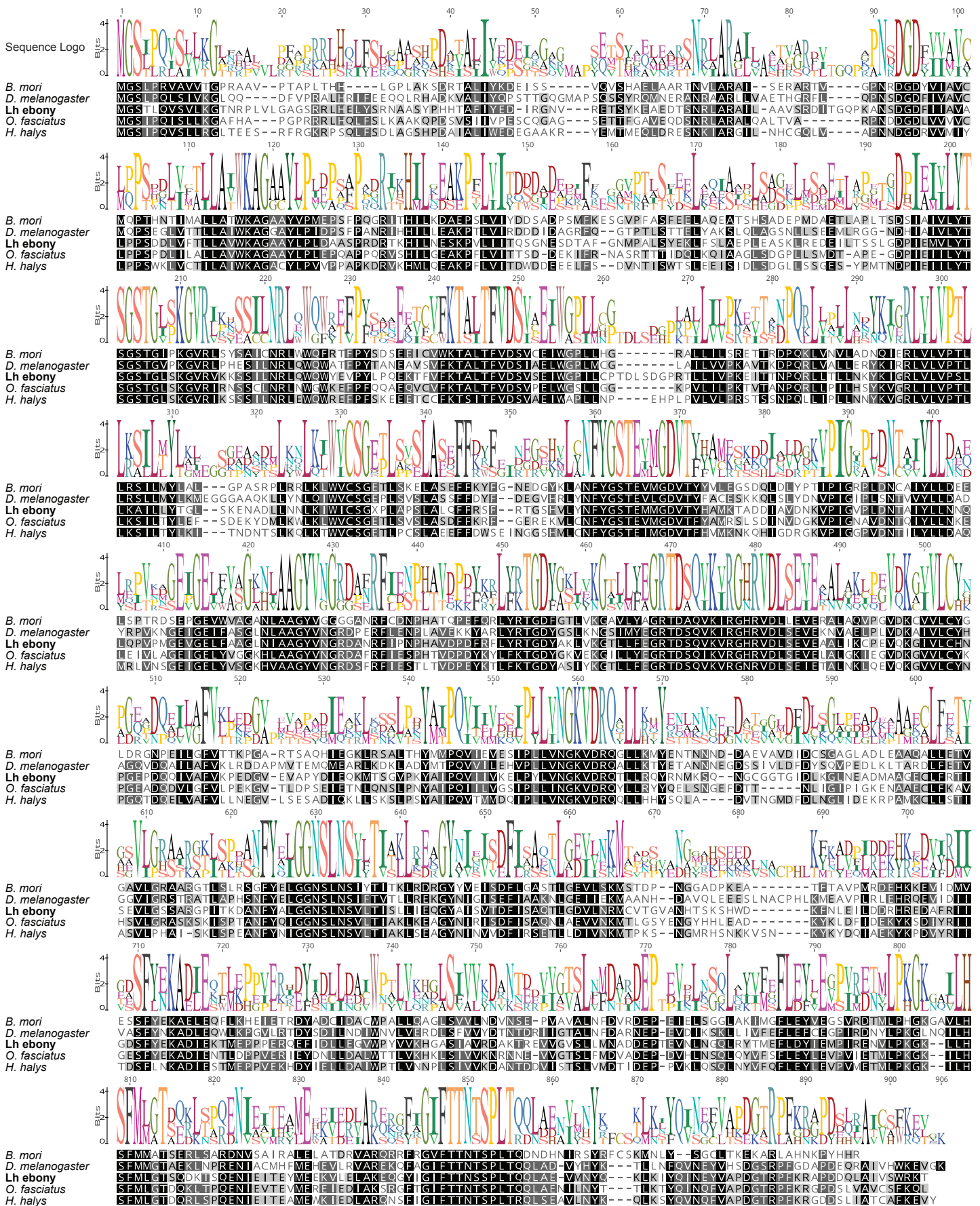

Supplement: Supplementary file 1 [file insects-13-00986-s001.zip › Figure S3 ebony.pdf]

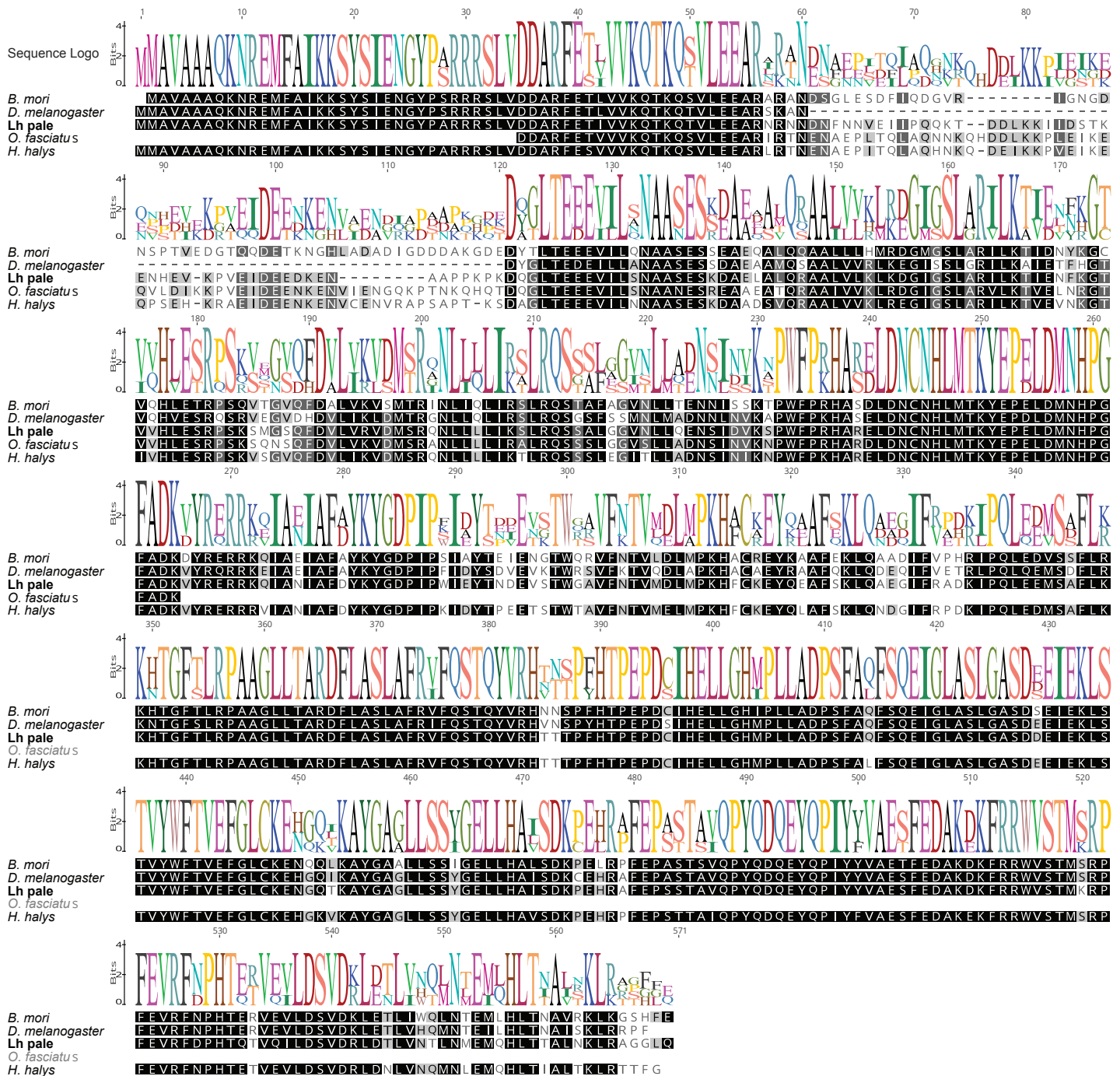

Supplement: Supplementary file 1 [file insects-13-00986-s001.zip › Figure S4 pale.pdf]

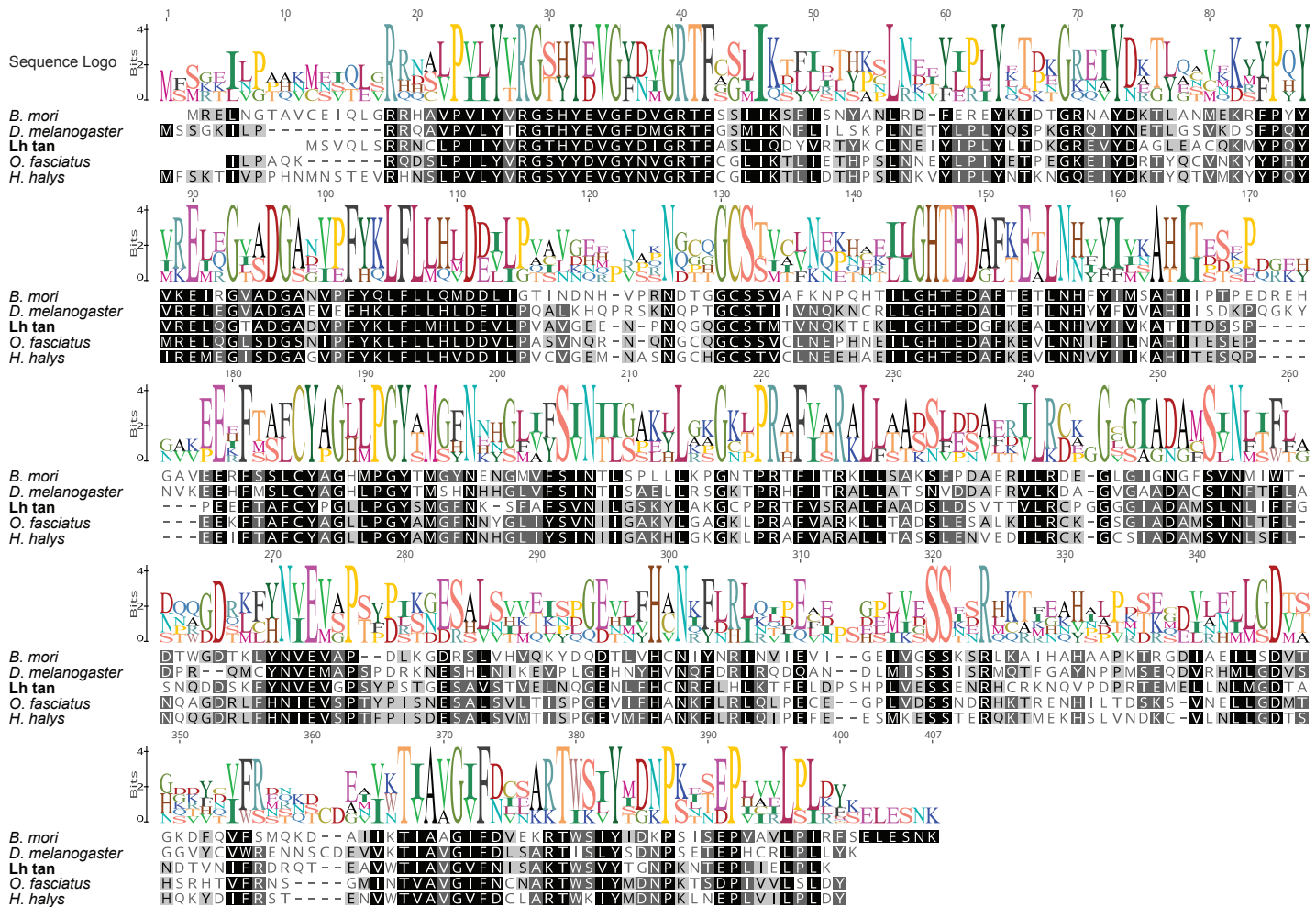

Supplement: Supplementary file 1 [file insects-13-00986-s001.zip › Figure S5 tan.pdf]

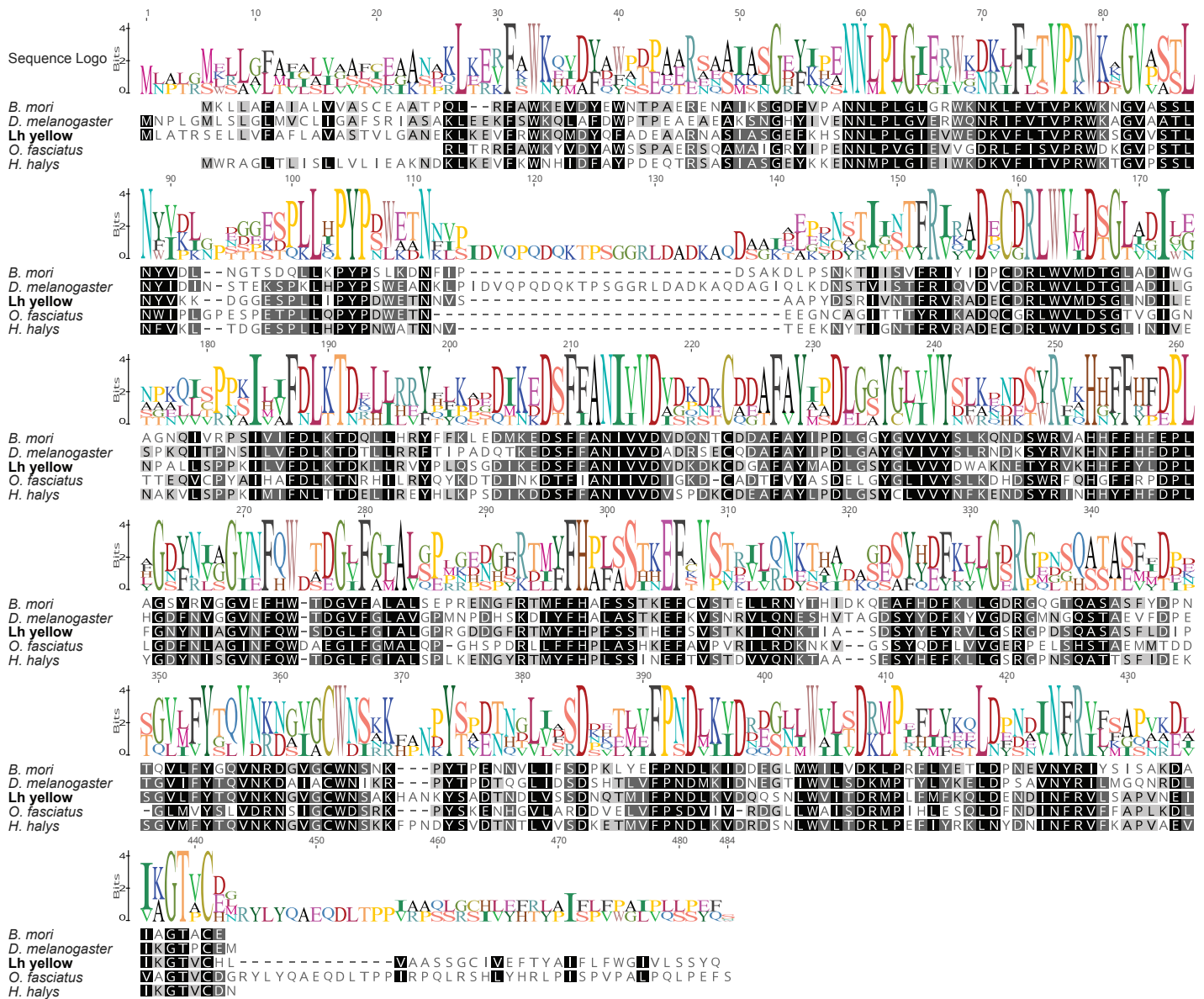

Supplement: Supplementary file 1 [file insects-13-00986-s001.zip › Figure S6 yellow.pdf]

- Coleoptera
- Diptera
- Hemiptera
- Hymenoptera
- Lepidoptera

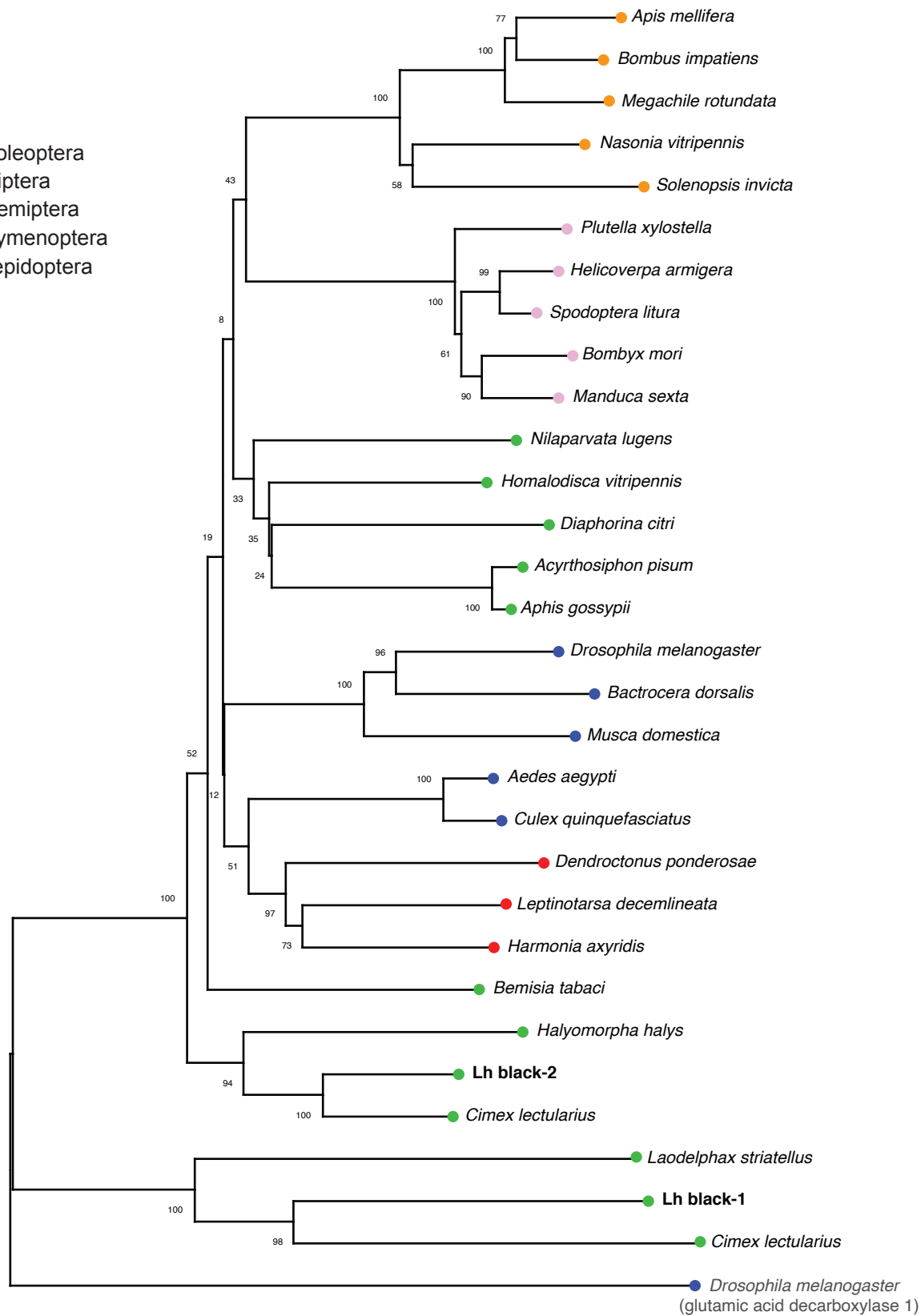

Supplement: Supplementary file 1 [file insects-13-00986-s001.zip › Figure S7 NJ phylogeny.pdf]
